# A developmental insurance policy

**DOI:** 10.7554/eLife.26260

**Published:** 2017-03-28

**Authors:** Nestor Saiz, Anna-Katerina Hadjantonakis

**Affiliations:** Sloan Kettering Institute, Memorial Sloan Kettering Cancer Center, New York, United States; Sloan Kettering Institute, Memorial Sloan Kettering Cancer Center, New York, United StatesHadjanta@mskcc.org

**Keywords:** inner cell mass, trophectoderm, cell fate, preimplantation, cell plasticity, Mouse

## Abstract

Why does a totipotent state linger within the inner cell mass of mouse embryos?

**Related research article** Posfai E, Petropoulos S, de Barros FR, Schell JP, Jurisica I, Sandberg R, Lanner F, Rossant J. 2017. Position- and Hippo signaling-dependent plasticity during lineage segregation in the early mouse embryo. *eLife* **6**:e22906. doi: 10.7554/eLife.22906

The very first decisions in the life of a mammal are made even before the embryo implants into the womb. During this time, as the number of cells in the embryo increases from one to two to four and so on, the cells start to specialize to form distinct lineages. The first choice a cell faces is whether to join a cell population called the inner cell mass and become part of the embryo, or to join the trophectoderm lineage and become part of the placenta.

The biology of this cell fate decision has been a subject of intrigue and experimental pursuit for over half a century. Building on landmark work by the late Krystof Tarkowski, Martin Johnson and others (Johnson and Ziomek, 1981; Tarkowski and Wróblewska, 1967), recent studies have demonstrated the importance of the Hippo signaling pathway – a pathway well known for regulating cell growth and death – in this process (reviewed in Sasaki, 2017). These studies have established how the polarity and position of a cell either cause activation of the Hippo pathway in the inner cells of the embryo, or inhibit it in the outer cells of the embryo to promote the expression of genes encoding a trophectoderm identity.

Previous attempts to determine the exact timing of when cells commit to either the inner cell mass (ICM) or the trophectoderm (TE) lineage yielded somewhat conflicting results. Now, in eLife, Janet Rossant and colleagues – including Eszter Posfai of the Hospital for Sick Children in Toronto as first author – report how they have used the thread and needle of fluorescent reporters and single-cell transcriptomics to stitch together classic and recent findings on this topic (Posfai et al., 2017).

A transcription factor called CDX2 has a central role in triggering the TE transcriptional program. The expression of CDX2 in cells that go on to become part of the TE relies on a complex called TEAD-YAP, which is activated by inhibition of the Hippo pathway in the outer cells of the embryo (Nishioka et al., 2009). Posfai et al. used a CDX2-GFP fusion (McDole and Zheng, 2012) to sort CDX2-positive and CDX2-negative cells, followed by single-cell RNA sequencing, to determine how the TE and ICM transcriptional programs became established as the embryo developed from the 16-cell stage to the 32-cell stage.

These data raise the question of what the progressive stabilization of cell fate might tell us about commitment to either lineage. Could the expression of TE genes restrict cells to a TE fate even when challenged experimentally (i.e., when placed in a new context)? In the assays used to test these questions, either single cells have to be implanted into genetically-distinct host embryos to generate a chimera, or an embryo needs to be rebuilt from isolated cells of one particular type (inner or outer). The contribution of daughter cells to the resulting embryo will reveal details about lineage commitment in the parental cells. With these techniques, the labs of Tarkowski, Johnson and Rossant previously established that both the inner and outer cells remain totipotent – that is, they can give rise to the ICM and TE lineages – until the 16-cell stage, with some inner cells remaining totipotent until the 32-cell stage (Suwińska et al., 2008; Rossant and Vijh, 1980; Ziomek et al., 1982).

Posfai et al. perform a contemporary version of these classic experiments using the CDX2-GFP fluorescent genetic marker, rather than cell position, to discriminate between prospective TE and ICM cells. They were able to precisely match cell fate commitment to the relevant gene expression profile of individual cells for both (GFP-positive and GFP-negative) populations at successive stages of development. This allowed them to confirm previous results and to paint a detailed picture of the molecular players that are potentially involved in stabilizing these two cell fates. For cells expressing CDX2, cell fate is basically sealed soon after blastocyst formation (at the 32-cell stage), and they are unable to give rise to the ICM. ICM cells, on the other hand, delay their commitment by an additional cell cycle, up until some point between the 32- and 64-cell stages (Figure 1). Posfai et al. confirm these results by genetically and pharmacologically modulating the activity of the Hippo pathway, which connects apico-basal cell polarity (or the lack of it in ICM cells) to gene expression.

**Figure 1.**
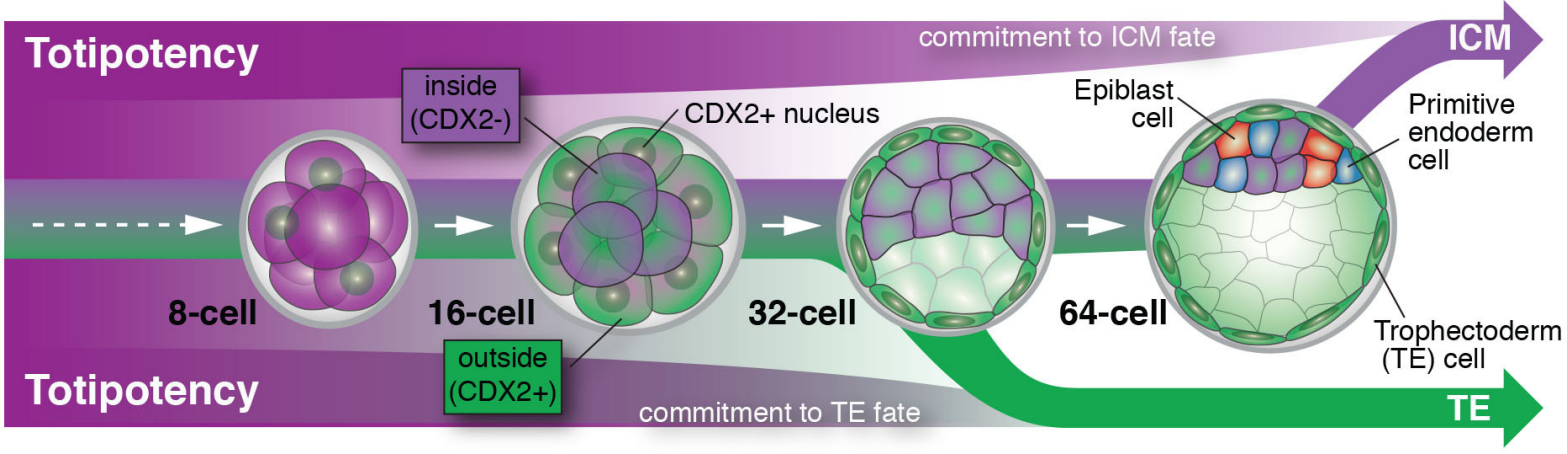
Cell differentiation in mammalian embryos. Cells that develop into the trophectoderm (TE) express the transcription factor CDX2 (denoted here as CDX2+) and commit to their cell fate at around the 32-cell stage. Cells that will develop into the inner cell mass (ICM) keep their options open and only commit to their cell fate at a time between the 32- and 64-cell stage; these cells do not express CDX2 (denoted here as CDX2-). As embryos grow from the 32- to the 64-cell stage, ICM cells start to differentiate into two new cell lineages: the embryonic epiblast (future fetus) and the extra-embryonic primitive endoderm (future yolk sac).

This raises the question of why commitment to the ICM lineage takes place later during development. Perhaps it is no coincidence that ICM cells gradually begin to make their next cell fate choice during this time window. It is at this time that ICM cells make a decision to become epiblast (future fetus) versus primitive endoderm (future yolk sac). Therefore, the ICM may not be a cell fate per se, but rather a transitory state that lasts only until all the cells in the embryo have been allocated to one of the three lineages that make up the blastocyst. An asynchrony in making these early fate decisions could therefore reflect a developmental insurance policy: a strategy to guarantee that enough cells differentiate for each of the cell types that lay the foundation for all embryonic and extra-embryonic tissues (Saiz et al., 2016).
